# Lateral Preference and Inter-limb Asymmetry in Completing Technical Tasks During Official Professional Futsal Matches: The Role of Playing Position and Opponent Quality

**DOI:** 10.3389/fpsyg.2021.725097

**Published:** 2021-08-19

**Authors:** Luiz H. Palucci Vieira, Carlos A. Kalva-Filho, Felipe B. Santinelli, Filipe M. Clemente, Sergio A. Cunha, Caroline V. Schimidt, Fabio A. Barbieri

**Affiliations:** ^1^Human Movement Research Laboratory (MOVI-LAB), Graduate Program in Movement Sciences, Department of Physical Education, School of Sciences, São Paulo State University (UNESP), Bauru, Brazil; ^2^Escola Superior Desporto e Lazer, Instituto Politécnico de Viana do Castelo, Rua Escola Industrial e Comercial de Nun'Álvares, Melgaço, Portugal; ^3^Sports Assessment Laboratory, Faculty of Physical Education, State University of Campinas, Campinas, Brazil

**Keywords:** skill-related performance, notational analysis, game activities, indoor football, contextual factors, motor control

## Abstract

This study had the purpose of analyzing dominant and non-dominant limb performances (frequency of use and accuracy) during match-play technical actions with ball possession (receiving, passing, and shooting a ball) in professional futsal and also check for the possible influence of playing position and the quality of opponent. We have analyzed data pertaining to eight matches of the FIFA Futsal World Cup Thailand 2012™ in which 76 male professional senior futsal players participated (44 right-footed and 32 left-footed). In total, we coded 5,856 actions (2,550 ball receptions, 3,076 passes, and 230 shoots). Our main findings were that (a) players used the dominant limb more frequently than the non-dominant limb for all actions considered [*p* < 0.001; effect size (ES) medium-to-large]; (b) accuracy was generally greater when using the dominant limb, regardless of the quality of opponent (*p* < 0.01; ES large); and (c) in shooting actions, pivots showed similar accuracy between dominant and non-dominant limbs (*p* = 0.51; ES small). The study suggested that when completing technical actions with the ball in futsal, high-level players depended to a greater extent on the use of their dominant lower limb during official matches. Excepting a similarity detected between limbs on shooting performance of pivots, players from all positional roles generally showed a higher accuracy rate in receiving, passing, and shooting a ball when using their dominant limb as compared to their non-dominant one during match-play and the limb usage and accuracy seemed to be independent of the quality of opponents.

## Introduction

Futsal is characterized as an open team sport (Correa et al., 2012, 2016) in which there are constant game-related technical-tactical actions such as interactions of the player with ball possession. In general, each futsal player must receive and perform passes at a pace of at least 2 passes/min during official matches (Vieira et al., 2016b), resulting in team totals of ~550 ball receptions, 200 passes, and 26 shots per game (Dogramaci et al., 2015b; Yiannaki et al., 2020). Accurate execution of these game actions was found to be critically important during offensive sequences that ended in shots and goal scoring (Barbieri et al., 2010; Lapresa et al., 2013; Sarmento et al., 2016). Higher skill-related accuracy (e.g., passing and shooting) and greater playing time with ball possession require efficient ball manipulation skills with both lower limbs, and these skills generally discriminate winning from drawing and losing peers (Moura et al., 2014; Dogramaci et al., 2015a; Balyan and Vural, 2018).

Given possible player and team discrepancies in the capacity to control right and left-sided body movements (Teixeira et al., 2003; Haywood and Getchell, 2009), limb dominance defined as limb preference when completing a given movement (task) can be important (Grouios, 2004). Symmetry has been generally recognized as an agreement or absence of statistically significant differences in movement outputs between right and left lower limbs, while asymmetry is assumed when right or left limb use has incongruent results (Sadeghi et al., 2000). In one of the first investigations aimed at understanding dominance and symmetry/asymmetry effects on match-play technical performance, Carey et al. (2001) found that soccer players were proportionally right-footed to the same degree as the general population, with 80–85% showing a foot preference for completing several game actions (e.g., receiving, passing, and shooting) but without differences on accuracy rates between dominant and non-dominant limbs and in left- and right-footed player comparisons. The results of such investigations, using data derived from the FIFA 1998 World Cup, make it uncertain whether long-term practice might lead to symmetrical, two-footed players (Carey et al., 2001). In another research, general motor ability has been shown to be similar among individuals with a distinguishable (i.e., right or left) foot dominance (Way, 1958). These studies provide examples that contradict the assumption that left (vs. right) limb preference might be linked with more skilled sports performance (Grouios, 2004), particularly in football codes.

A factor relevant to futsal performance is that futsal players perform many more technical actions during a game than soccer players (Andrade et al., 2015; Vieira et al., 2016a; Yiannaki et al., 2018). Thus, studies based on soccer players may not be pertinent to the playing characteristics of futsal players. Importantly, futsal players also perform a substantial amount of match-play activity at high speed (Barbero-Alvarez et al., 2008; Vieira et al., 2017; Palucci Vieira et al., 2021), increasing overall game demands and constraining athletes to avoid delayed decision-making and engage in quick and complete ball possession technical actions (Duarte et al., 2009; Gómez et al., 2015b; Balyan and Vural, 2018; Yiannaki et al., 2018). Considering the high-pressure and short time and space for futsal decision-making, futsal players may often be forced to use both dominant and non-dominant limbs during the game. For example, shooting a rolling ball after a pass is a more frequent action than shooting a stationary ball (Barbieri et al., 2010), and it is not unusual for a futsal player to perform a pass that directly transfers the ball to a teammate without time to control or otherwise manipulate the ball (Sarmento et al., 2016). These examples provide insights against a possible predominant use of the dominant limb/foot during competitive futsal matches. To our knowledge, past studies of game-related technical performance in futsal have almost exclusively relied upon computations of the absolute frequency and accuracy of match skills without considering whether the dominant or non-dominant lower limb was used, or where the action was located on the court (Dogramaci et al., 2015a,b; Vieira et al., 2016b; Barth et al., 2019; Méndez et al., 2019). There was just one recent exception that addressed the limb laterality question in a single reference team, with findings indicating that over 80% of futsal ball receptions and passes were completed using the dominant foot and percentage of successful passes did not differ between limbs but with no information, to date, on shooting (Yiannaki et al., 2020). Thus, the current evidence on the frequency of use in addition to performance of the dominant vs. non-dominant limb is merited to be tested also on a large sample in order to check whether it shows replicability.

Finally, the prominent influence of positional role and the quality of opponents were not taken into account as potential confounders in research investigating asymmetries during match-play technical actions. Indeed, the need to consider position- and opponent-related constraints when interpreting match data like notational indicators is highlighted in existing reviews (Gómez et al., 2013; Mackenzie and Cushion, 2013; Sarmento et al., 2014; Aquino et al., 2017b). Depict performance as a function of the context it is happening seems the main reason supporting the use of these independent measures. In general, low- or middle-ranked opponents may induce reference athletes outperforming technical tasks (passing and shooting accuracy) (Varley et al., 2017) while attacking players have the most unstable proficiency (e.g., possessions won) (Bush et al., 2015). Preliminary research specifically during futsal game-play has also shown that such factors may have significant effects on the behavior of player (Travassos et al., 2011; Gómez et al., 2019; Méndez-Domínguez et al., 2019). On the other hand, the possibility of rotating positions across the game can make unsure whether playing position is a critical modulating component to in-game movement outputs (Spyrou et al., 2020) while the quality of opponent produced only slight effects in a study assessing their influence on the outcome of attacks in elite futsal match-play (Gómez et al., 2019).

Here, we aimed to analyze player use, court location, and performance with dominant and non-dominant limbs during match-play technical actions with ball possession (i.e., receiving, passing, and shooting) in official professional futsal tournament while checking the possible influence of playing position and quality of opponent. We further sought to compare the technical skill performances of right- and left-footed futsal players. Based on the previous studies (Carey et al., 2001; Lapresa et al., 2013; Barbieri et al., 2015; Sarmento et al., 2016; Varley et al., 2017), we hypothesized that futsal players would show similar frequencies of dominant and non-dominant limb use in receiving, passing, and shooting a ball, but accuracy, when completing these tasks, would be lower when players used the non-dominant limb. In addition, considering that actions more distant from the offensive goal have a lower likelihood of resulting in a score, we expected that there would be more accurate (vs. inaccurate) non-dominant limb actions farther from the goal. However, we also hope that these outcomes may be dependent upon playing position [e.g., players pertaining to attacking (more risk) role showing similar frequency of use but less consistent performance between lower limbs] and the quality of the opponent (e.g., poor-ranked peers inducing reference players performing in-game technical tasks with a lower inter-limb asymmetry). Finally, we hypothesized that right- and left-footed players would not differ in their use of dominant and non-dominant limbs during futsal actions.

## Materials and Methods

### Subjects

This study involved 76 (44 right-footed and 32 left-footed) male professional senior outfield futsal players who had participated in the FIFA Futsal World Cup Thailand 2012™. We analyzed their actions with the ball in eight matches, mainly during the play-off stage (seven matches: Argentina vs. Serbia [*N* = 9], Brazil vs. Colombia [*N* = 10], Brazil vs. Spain [*N* = 10], Spain vs. Russia [*N* = 11], Italy vs. Colombia [*N* = 9], Portugal vs. Paraguay [*N* = 8], and Russia vs. Czech Republic [*N* = 10]). One match involved a qualifier, rather than a play-off game (Portugal vs. Japan [*N* = 9]). We analyzed actions of all the outfield players participating in such games, except that we excluded four play actions from the initial sample (*N* = 80 players) in which there were an insufficient number of technical events performed (as seen in criteria below). A decision was made to select most knockout stage matches given their possible higher demands related to ball possession as compared to group stage fixtures (e.g., Ismadi Ismail and Nunome, 2021). Matches were played on official standard futsal courts (40 m × 20 m), and the rules adopted were specified in FIFA^®^ Regulations document (FIFA, 2012b). We obtained prior permission to complete this study from our local University Human Research Ethics Committee–Instituto de Biociências, Campus de Rio Claro, SP, Brazil (protocol #4842).

### Data Acquisition and Coding

We monitored all matches using publicly available broadcast TV footage and data retrieved from official reports of the competition (FIFA, 2012a). We used these data to help compute match-related technical variables and define the dominant and non-dominant limb for each player. A single experienced examiner identified actions performed with the ball by each of the included players and coded these actions, using notational analysis in the Skout^®^ software (Barros et al., 2008). The annotated technical tasks were receiving, passing, and shooting a ball when performed solely with lower limb segments of either limb (but not when additional body regions such as the trunk and head, allowed by the rules, were used). With minor adaptations borrowed from previous studies (Dogramaci et al., 2015b; Sarmento et al., 2016; Yi et al., 2019), we adopted the following definitions of technical tasks:

- Receiving: all situations in which a player received the ball in play delivered from a teammate, then cushioned it and was able to perform an ensuing game action, with receiving accuracy considered when ball possession was maintained after receiving the ball.
- Passing: all situations in which there was a ball touch with the intention of delivering it to a given teammate, with accuracy considered when the ball was successfully received by a teammate (i.e., completed passes), irrespective of subsequent events.
- Shooting: all attempts to score a goal, with accuracy, considered when the ball reached the target.

**Figure 1:**
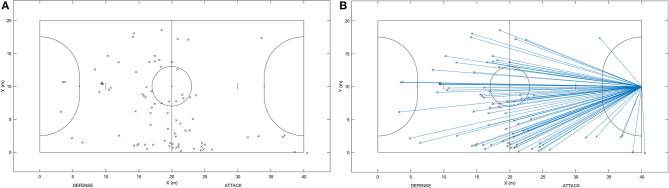
Example of **(A)** technical actions of players recorded across the court (“x” symbol represents the estimated location where a given category of technical actions occurred [e.g., accurate passing with dominant limb]—see *Data acquisition and coding* for more information) and **(B)** illustration of lines drawn connecting location where each of technical actions occurred to the midpoint of the opposition goal line, used to measure the Euclidean distance between them.

We identified a total of 5,856 technical actions across matches that were included. Of these, there were 2,550 ball receptions, 3,076 passes, and 230 shots. These actions were coded continuously during the whole match as to (a) whether players used their dominant or non-dominant lower limbs, (b) whether players were accurate or inaccurate in their proficiency, and (c) a miniaturized field was used to visually estimate (similar to manual tracking features) the bi-dimensional (2D) position values on the *x*- and *y*-axis occurred for each of the actions (i.e., a campogram; Barros et al., 2008). Figure 1A shows an illustration of these positional values. A test-retest protocol (using ~12% of the processed games randomly selected) interspaced by >30 days was performed to determine the intra- (i.e., the same examiner performed measurements twice) and inter-reliability of the observer (i.e., a second examiner analyzed the same video sequences). The reliability was good for the study [intraclass correlation coefficient (ICC) intra-observer reliability = 0.87–0.92 and ICC inter-observer reliability = 0.75–0.88].

Past literature has suggested that at least 20 match-based technical actions using both lower limbs are required in order to be able to distinguish performance between dominant and non-dominant sides of the body (Carey et al., 2001). Thus, four of the players from the initial sample (*N* = 80) were excluded from the final analysis due to participation in an insufficient number of events. The criteria, as previously defined, were used to identify limb dominance of players when, after pooling all of their actions, it became clear that they performed ≥50% of their actions while using a given limb (Carey et al., 2001). To confirm whether each athlete preferred to play using their left- or right-foot, we also checked information about them from another public repository of their anthropometric characteristics (www.playmakerstats.com/). Finally, players were also sub-grouped according to the positional role as a defender (*N* = 27), winger (*N* = 36), or pivot (*N* = 13) (Illa et al., 2021) and based on the final classification of the opponent team (i.e., ranking). To compute this later index, we applied the K-means cluster analysis (Gómez et al., 2011, 2015a; Aquino et al., 2017a) to the cumulative sum of points earned by each team across the matches. Three clusters were identified taking into account all the national squads participating in the tournament (“top-ranked” [1st−3rd position], “middle-ranked” [4th−10th position], or “bottom-ranked” [11th−24th position]). Considering the sample/main phase of the tournament included here, only the first two clusters of rankings were considered as an independent measure.

### Dependent Variables

Using specific Matlab software (MathWorks Inc., Natick, MA, USA), we computed the following dependent variables: (a) the absolute frequency of all executed technical actions, (b) the absolute frequency of accurate and inaccurate actions, (c) the percentage of accurate and inaccurate executions of technical actions, and (d) using 2D positional data, the Euclidean distance between the midpoint offensive goal line and the location where technical actions occurred for each category of coded actions (e.g., accurate passes completed using the dominant-limb; as shown in Figure 1B). To obtain positional data, we converted the pixel values outputted by the Skout^®^ program into real coordinates, using the width and length of the court as reference parameters.

### Statistical Analysis

As Shapiro-Wilk's test indicated that most variables were non-normally distributed, the results were described as median, interquartile range, and extreme values (i.e., minimal and maximal). We compared left-footed and right-footed players using Mann-Whitney's test, and we used Friedman's test to compare performance between the dominant and non-dominant limbs of players, based on the total number of technical events (i.e., accurate or inaccurate) and their percent accuracy. When necessary, we applied Wilcoxon's test to determine the significant differences between limbs and/or number of technical events. Considering that some actions were not performed by a specific player (i.e., different numbers of observations for each situation), we applied the Kruskal-Wallis test to compare the average distance of action's occurrence from the goal line, observing possible differences between right- and left-footed actions and whether actions were accurate or inaccurate. Finally, Mann-Whitney's test was also used to compare the frequencies of each action that occurred and the average distance from the goal line between top- and middle-ranked opponents. We calculated effect sizes (ES) for pairwise comparisons (ES = z.√n), and we classified these as negligible (<0.1), small (0.1–0.29), medium (0.3–0.49), and large (>0.5) (Fritz et al., 2012). We analyzed all data using the Statistical Package for Social Science software (SPSS Inc., Chicago, IL, USA) version 22.0, with the level of statistical significance set at *p* < 0.05.

## Results

Examining right-footed and left-footed players performance across pooled actions, we examined dominant and non-dominant limb use, first by considering the absolute number of technical actions performed (as shown in Table 1; Supplementary Table 1). The pooled total actions (accurate plus inaccurate) performed by the dominant limb (median (IQR) [min–max]; 62 (52) [1–177] actions) were higher (*p* = 0.001; ES = 0.86 [Large]) than non-dominant limb (13 (21) [0–77] actions). These differences were also observed for the pooled data on passes (dominant: 29 (30) [1–99]; non-dominant: 2 (4) [0–15]; *p* = 0.001; ES = 0.87 [Large]), shooting (dominant: 2 (3) [0–9]; non-dominant: 0 (1) [0–2]; *p* = 0.001; ES = 0.75 [Large]) and receptions (dominant: 24 (23) [9–73]; non-dominant: 3 (5) [0–26]; *p* = 0.001; ES = 0.86 [large]).

**Table 1:** Median, interquartile range, and extreme values regarding the frequency counts (i.e., absolute number; a.u.) of technical parameters performed using dominant and non-dominant limb.

	**Dominant limb**		**Non-dominant limb**		***p*-value**
	**Accurate**	**Inaccurate**	**Accurate**	**Inaccurate**	
Total	57 (52.5) [0–170]^*^	3 (3) [0–9]^*^	12 (21.5) [0–76]^*^	0 (1) [0–4]^*^	0.001
Total (%)	84.3 (7.9) [0–97]^*^	5 (4) [0–100]^*^	8.5 (8.4) [0–35]^*^	0 (1.4) [0–8.7]^*^	0.001
Passing	29 (30.5) [0–95]^*^	2 (2) [0–8]	2 (4) [0–14]	0 (1) [0–3]^*^	0.001
Shooting	1 (3) [0–7]^*^	1 (1) [0–4]^*^	0 (0) [0–1]	0 (0) [0–2]	0.001
Ball reception	24 (22.5) [0–72]^*^	0 (1) [0–2]^*^	7 (20) [0–61]^*^	0 (0) [0–1]^*^	0.001

*p-value: statistics significance observed through Friedman's test*.

* *Significant difference from all other categories (accurate or inaccurate) observed through Wilcoxon's test (p < 0.05)*.

Both dominant and non-dominant limbs presented a higher number of accurate vs. inaccurate actions (*p* < 0.001; ES > 0.61). The number of accurate actions using the dominant limb was higher than the number of accurate actions using the non-dominant limb (*p* < 0.01; ES > 0.72). The number of accurate passes with the non-dominant limb tended to be more frequent than the number of inaccurate passes with the dominant limb but this difference was not statistically significant and was accompanied by negligible ES (*p* = 0.06; ES = 0.04). There were no significant differences observed between accurate and inaccurate shooting frequencies using the non-dominant limb (*p* = 0.98; ES = 0.01). Receiving a ball occurred more frequently with the dominant than the non-dominant limb (*p* < 0.01; ES > 0.21).

**Figure 2:**
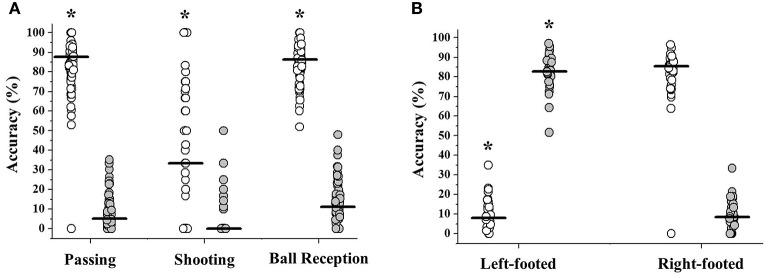
Accuracy values observed for technical actions completed using **(A)** the dominant (open symbols) or non-dominant limb (gray symbols) in all players pooled. *Significant differences between limbs observed through Wilcoxon's test (*p* < 0.05) and **(B)** separately for left-footed and right-footed players using the right limb (open symbols) or left limb (gray symbols). *Significant differences between groups using the same limb observed through Mann-Whitney's test (*p* < 0.05). Horizontal lines represent the group median values.

Accuracy was higher with the dominant vs. non-dominant limb for passing (*p* < 0.01; ES = 0.75), shooting (*p* < 0.01; ES = 0.46), and receiving a ball (*p* < 0.01; ES = 0.75) (as shown in Figure 2A). Supplementary Table 1 contains the results of positional data calculations and analyses. The average distance between the offensive goal midpoint line and the location where the passing, shooting, or receptions occurred did not differ as a function of either limb dominance (*p* = 0.16; ES = 0.01) or action accuracy (*p* = 0.18; ES = 0.01).

Comparative analyses between left-footed and right-footed players are presented in Table 2. The total number of pooled actions performed was not different between left-footed and right-footed players (*p* = 0.63). Both right-footed and left-footed players presented a higher number of accurate and inaccurate actions using their dominant vs. their non-dominant limb (*p* < 0.001; ES: Medium to Large). Dominant limb superiority was also observed for accuracy vs. inaccuracy values (*p* < 0.001; ES > 0.42) (Figure 2B).

**Table 2:** Median, interquartile range, and extreme values of frequency counts (absolute number; a.u.) of technical parameters presented by left-footed and right-footed players.

	**Left-footed**	**Right-footed**	**ES [Rating]**
	**(*n* = 32)**	**(*n* = 44)**	
Total actions	66.5 (55) [13–174]	70 (65) [1–198]	0.01 [Negligible]
Accurate—RL	6 (7.8) [0–37]	57 (47) [0–170]^*^	0.58 [Large]
Inaccurate—RL	0 (1) [0–4]	3 (2) [0–9]^*^	0.47 [Medium]
Accurate—LL	57 (54.8) [11–137]	5 (8.5) [0–31]^*^	0.67 [Large]
Inaccurate—LL	4 (3.8) [0–9]	0 (1) [0–3]^*^	0.55 [Large]
Passing			
Accurate—RL	2 (4) [0–14]	28 (27) [0–95]^*^	0.62 [Large]
Inaccurate—RL	0 (1) [0–3]	2 (2) [0–8]^*^	0.37 [Medium]
Accurate—LL	29.5 (30) [4–78]	2 (4) [0–13]^*^	0.65 [Large]
Inaccurate—LL	2 (3) [0–7]	0 (1) [0–2]^*^	0.38 [Medium]
Shooting			
Accurate—RL	0 (0) [0–1]	1 (3) [0–7]^*^	0.23 [Small]
Inaccurate—RL	0 (0) [0–1]	1 (1) [0–3]^*^	0.16 [Small]
Accurate—LL	1 (3) [0–5]	0 (0) [0–1]^*^	0.34 [Medium]
Inaccurate—LL	1 (1) [0–4]	0 (0) [0–2]^*^	0.22 [Small]
Ball reception			
Accurate—RL	3 (5) [0–23]	24 (21) [0–72]^*^	0.53 [Large]
Inaccurate—RL	0 (0) [0–1]	0 (1) [0–2]^*^	0.13 [Small]
Accurate—LL	24.5 (26) [6–61]	3 (5) [0–19]^*^	0.66 [Large]
Inaccurate—LL	0 (1) [0–2]	0 (0) [0–1]^*^	0.14 [Small]

*ES, effect size; LL, Left limb; RL, Right limb;*

* *Significant differences between groups observed through Mann-Whitney's test (p < 0.05)*.

The analyses according to positional role of players were presented in Table 3 and Supplementary Table 2. Wingers presented higher frequencies of success using dominant limb for all technical actions (*p* < 0.01; ES > 0.38), while the lowest frequencies were observed on unsuccessful actions using the non-dominant limb (*p* < 0.01; ES = 0.87). Defenders and pivots presented higher frequencies of success using the dominant limb to passing (*p* < 0.01; ES > 0.59), ball reception (*p* < 0.01; ES > 0.63), and total of actions (*p* < 0.01; ES = 0.78). These positions of the player presented the lower frequencies of unsuccessful for shooting using the non-dominant limb (*p* < 0.01; ES > 0.60). In addition, lowest frequencies of unsuccessful passing using the dominant limb were presented by pivots to passing (*p* = 0.03; ES = 0.74) and by defenders to receive the ball (*p* = 0.01; ES = 1.21).

**Table 3:** Median, interquartile range, and extreme values regarding the frequency counts (i.e., absolute number; a.u.) of technical parameters using dominant and non-dominant limb, computed according to positional role.

	**Dominant limb**		**Non-dominant Limb**		***p*-value**
	**Accurate**	**Inaccurate**	**Accurate**	**Inaccurate**	
Defenders					
Passing	33.5 (27) [6–72]^*^	2 (2) [0–6]	2 (5) [0–11]	0 (0) [0–3]^*^	0.01
Shooting	1 (2) [0–6]	1 (1) [0–3]	1 (1) [0–3]	0 (0) [0–2]^*^	0.01
Ball reception	26 (19) [3–58]^*^	0 (0) [0–1]^†^	3 (3) [0–14]^*^	0 (0) [0–1]	0.01
Total	65 (36) [9–133]^*^	3 (3) [0–8]^*^	5 (8) [0–22]^*^	0 (1) [0–3]^*^	0.01
Wingers					
Passing	30 (33) [0–95]^*^	2 (3) [0–8]	2 (4) [0–14]	0 (1) [0–3]^*^	0.01
Shooting	1 (1) [0–5]^*^	1 (1) [0–4]	1 (1) [0–4]	0 (0) [0–2]^*^	0.01
Ball reception	25 (29) [0–72]^*^	0 (1) [0–2]^*^	4 (7) [0–26]^*^	0 (0) [0–1]^*^	0.01
Total	58 (63) [0–170]^*^	4 (4) [0–9]^*^	6 (10) [0–37]^*^	0 (1) [0–4]^*^	0.01
Pivots					
Passing	19 (31) [4 −56]^*^	1 (2) [0–3]^†^	2 (3) [0–13]	0 (1) [0–2]^*^	0.01
Shooting	1 (3) [0–7]	1 (2) [0–3]	1 (2) [0–3]	0 (0) [0–1]^*^	0.02
Ball reception	18 (26) [5–43]^*^	1 (1) [0–2]^*^	4 (5) [0–6]^*^	0 (0) [0–1]^*^	0.01
Total	42 (57) [9–102]^*^	3 (3) [0–5]^*^	6 (6) [0–19]^*^	0 (1) [0–3]^*^	0.01

*p-value: statistics significance observed through Friedman's test*.

* *Significant difference from all other categories (accurate or inaccurate) observed through Wilcoxon's test;*

† *Difference between position observed through Mann-Whitney's test (p < 0.05)*.

**Figure 3:**
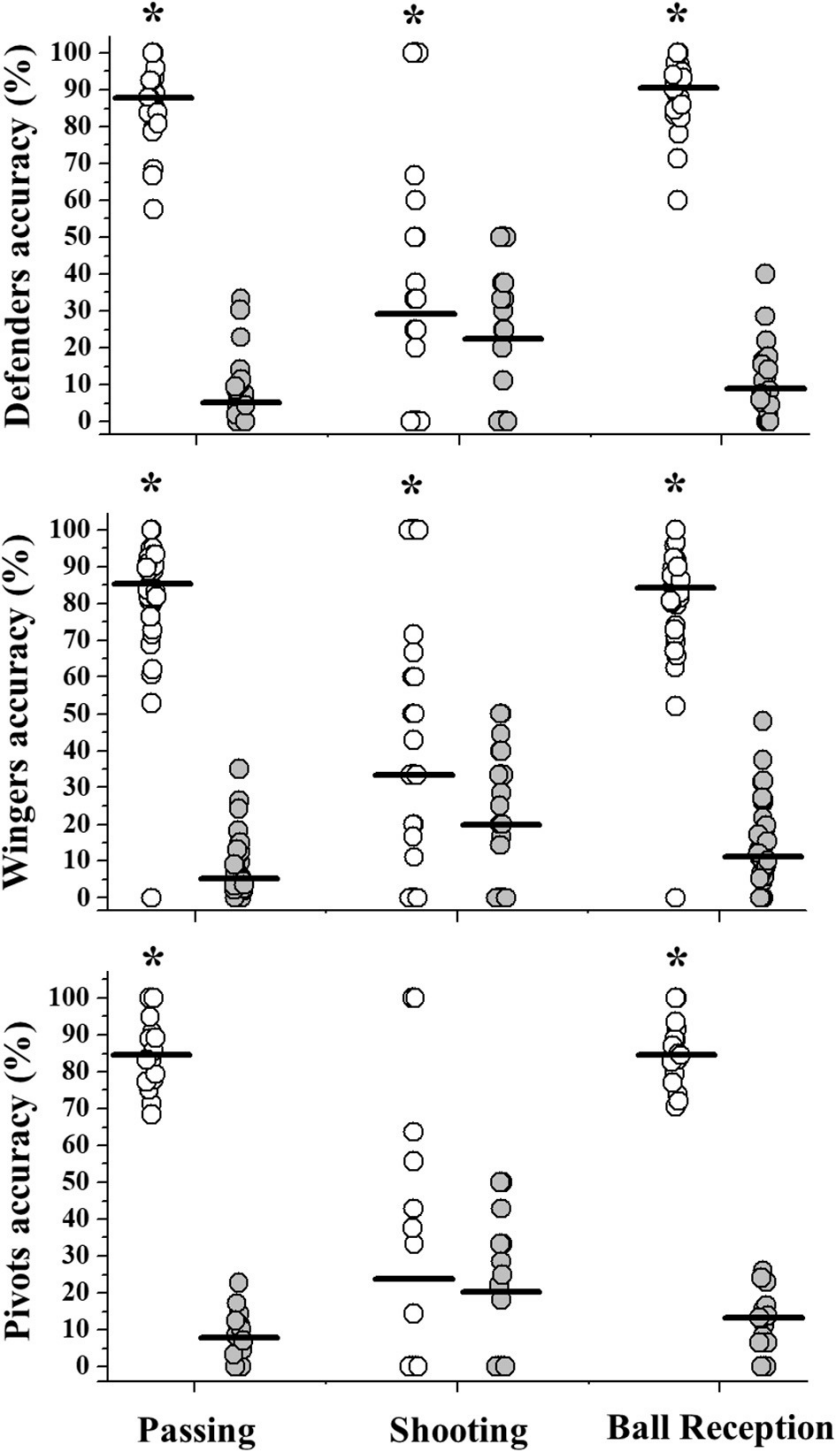
Accuracy (%) values observed for defenders, wingers, and pivots using the dominant (open symbols) or non-dominant limb (gray symbols). Horizontal lines represent the group median values. *Significant differences between limbs observed through Wilcoxon's test (*p* < 0.05).

The average distance between the offensive goal midpoint line and the location where the passing, shooting, or receptions occurred did not differ as a function of player role (*p* > 0.16) (Supplementary Table 2). The accuracy of wingers and defenders were higher using the dominant limb for passing (*p* = 0.01; ES = 0.86 and *p* = 0.01; ES = 0.87, respectively), shooting (*p* = 0.01; ES = 0.40 and *p* = 0.02; ES = 0.43, respectively), and receiving the ball (*p* = 0.01; ES = 0.85 and *p* = 0.01; ES = 0.87, respectively). Differently, despite the higher accuracy using the dominant limb for passing (*p* = 0.01; ES = 0.87) and receiving the ball (*p* = 0.01; ES = 0.87), pivots presented similar accuracy between limbs for shooting (*p* = 0.51; ES = 0.16) (Figure 3).

The frequencies of technical actions/performance as a function of opponent quality were demonstrated in Table 4 and Supplementary Table 3. Regardless of the quality of opponent, the ball reception and the total actions were different in accordance of both dominant limb (*p* < 0.01; ES > 0.39) and accuracy (*p* < 0.01; ES > 39). Against top-ranked opponents, the unsuccessful shooting and passing actions with the non-dominant limb presented the lowest frequencies (*p* < 0.01; ES > 0.62). In fact, against top-ranked opponents, successful passing with the dominant limb were found to be the most frequent (*p* < 0.01; ES > 0.87). Against middle-ranked opponents, the most frequent successful shooting were using the dominant limb (*p* < 0.01; ES > 0.47). In addition, against middle-ranked opponents, lower frequencies were observed for successful passing and total actions using the non-dominant limb, in comparison to matches against top-ranked opponents (*p* < 0.01; ES > 0.31).

**Table 4:** Median, interquartile range, and extreme values regarding the frequency counts (i.e., absolute number; a.u.) of technical parameters using dominant and non-dominant limb, computed according to the level of opponent.

	**Dominant limb**		**Non-dominant limb**		***p*-value**
	**Accurate**	**Inaccurate**	**Accurate**	**Inaccurate**	
Top-ranked					
Passing	26 (27) [0–95]^*^	2 (3) [0–6]	0 (0) [0–3]	1 (3) [0–5]^*^	0.01
Shooting	1 (2) [0–4]	1 (2) [0–4]	1 (2) [0–4]	0 (0) [0–1]^*^	0.01
Ball reception	23 (22) [0–72]^*^	0 (1) [0–2]^*^	3 (4) [0–15]^*^	0 (0) [0–1]^*^	0.01
Total	50 (48) [0–170]^*^	4 (3) [0–8]^*^	5 (6) [0–24]^*^	0 (1) [0–3]^*^	0.01
Middle-ranked					
Passing	36 (31) [8–78]^*^	2 (2) [0–8]^*^	3 (6) [0–14]^*^^†^	0 (1) [0–3]^*^	0.01
Shooting	1 (3) [0–7]^*^	1 (1) [0–3]	1 (1) [0–3]	0 (0) [0–2]^*^	0.01
Ball reception	28 (24) [9–61]^*^	0 (1) [0–2]^*^	4 (5) [0–26]^*^	0 (0) [0–1]^*^	0.01
Total	63 (54) [18–137]^*^	3 (3) [0–9]^*^	8 (9) [0–37]^*^^†^	1 (1) [0–4]^*^	0.01

*p-value: statistics significance observed through Friedman's test*.

* *Significant difference from all other categories (accurate or inaccurate) observed through Wilcoxon's test;*

† *Difference between level of opponent observed through Mann-Whitney's test (p < 0.05)*.

**Figure 4:**
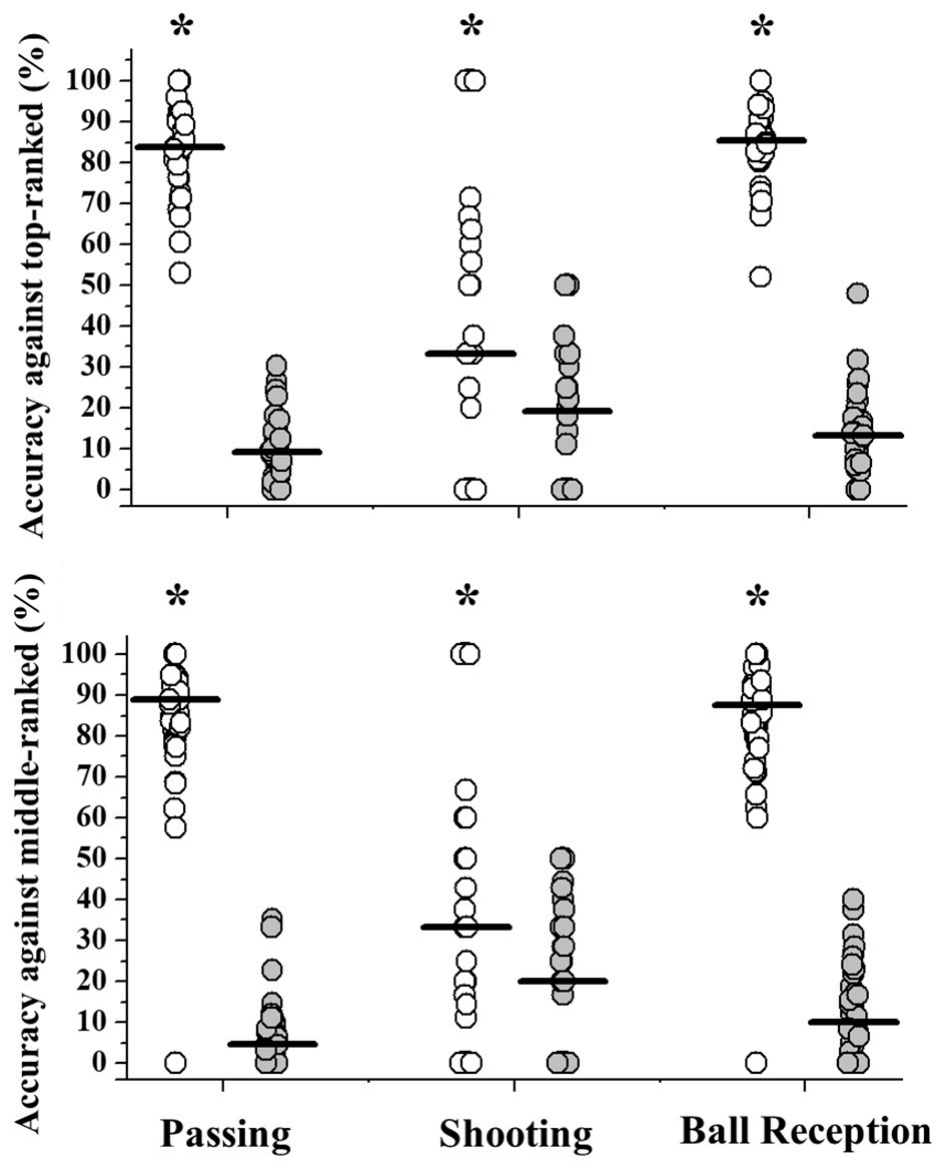
Accuracy (%) values observed according to opponent quality (top or middle-ranked) when using the dominant (open symbols) or non-dominant limb (gray symbols). Horizontal lines represents the group median values. *Significant differences between limbs observed through Wilcoxon's test (*p* < 0.05).

The average distance between the offensive goal midpoint line and the location where the receptions occurred was lower against middle-ranked opponents (*p* = 0.05; ES = 0.41) (Supplementary Table 3). The accuracy (Figure 4) for passing, shooting, and receiving the ball was higher using the dominant limb, regardless of the quality of the opponent (*p* < 0.03; ES > 0.36).

## Discussion

In this study, we tested whether futsal players have similar action frequencies and accuracies when receiving, passing, and shooting a ball with the dominant vs. non-dominant limbs, and we analyzed whether the distance of actions from the offensive goal would influence their accuracy with dominant vs. non-dominant limbs. In these analyses, we used data derived from an official tournament of elite futsal (a FIFA^®^ World Cup edition). We partly rejected our hypotheses, since the players used the dominant limb more frequently than the non-dominant limb in receiving, passing, and shooting a ball, and showed a higher accuracy rate when using their dominant limb. However, we verified that asymmetries in shooting performance were position-dependent, since players at forward position showed similar accuracy in this action for both limbs while it was not the case for defenders and wingers. Also, with few exceptions, the performance of dominant and non-dominant limbs during futsal actions was independent of the opponent quality and average distance between the locations where the actions with the ball occurred in relation to the goal midpoint line. Finally, in our test of whether the performance of dominant and non-dominant limbs during futsal actions was equal between right- and left-footed players, we generally confirmed our hypothesis. For all actions performed by the dominant limb, right- and left-footed players did not perform differently.

### Dominant vs. Non-dominant Limb Performance

Many great previous investigations determined possible differences in futsal techniques with dominant and non-dominant lower limbs, but these studies mainly tested kicking ability outside of match-play and in unopposed conditions. These studies have consistently shown poor accuracy and ball and foot velocities when using the non-dominant limb, across various age groups and playing standards (Barbieri et al., 2008, 2015; Teixeira and Teixeira, 2008; Vieira et al., 2016a; Palucci Vieira et al., 2019b). Other prior studies found that field test indices were not necessarily correlated with technical performance in match situations, resulting in limited ability to transfer earlier research findings to actual competitions (Ré et al., 2014; Serpiello et al., 2017). Indeed, one might argue that controlled field and/or laboratory studies have greater sensitivity for detecting skill execution and proficiency differences (Carling and Dupont, 2011). However, others have recognized that match play analyses aiming to record game-related skills of players are more suitable means of detecting player skills in need of improvement and contributing to coaching and staff plans for training sessions (Palucci Vieira et al., 2019a). To date, research has been unclear regarding whether the poorer performance using the non-dominant limb that was revealed in previous controlled studies may also occur in official futsal matches and the presented work was designed in an attempt to elucidate this question.

The number of actions and their accuracy were higher when players used their dominant vs. non-dominant limb, and it was independent of opponent quality. However, a position-dependent influence was noted regarding shooting outcomes, in which forwards but not defenders and wingers, demonstrated similar performance (i.e., accuracy) between limbs. It seems that the predominant attacking role of pivots, which also is generally occupying the central region of the court, may promote specific adaptations favoring their bilateral proficiency in shooting as compared to the remainder positional roles. Meanwhile, the situational variables (e.g., quality of opposition) have shown to modulate match-play technical performance in a range of indoor team sports (Sampaio et al., 2010; Gómez et al., 2013; Lago-Peñas et al., 2013), and our data also demonstrated a higher number of accurate passes as well as total accurate actions using the non-dominant limb when playing against middle-ranked teams. On the other hand, asymmetries persisted across all technical actions regardless of the quality of opponent. To date, only a few studies addressed the impact of quality of opponent in the effectiveness of technical actions with ball possession in futsal players. For example, while one existing work reported no effect exerted by the quality of opponent on futsal performance under habitual circumstances (Gómez et al., 2015b), significant influences of this contextual factor were found when there were dismissals of players in the matches (i.e., two yellow cards or a direct red card being received; Gómez et al., 2019). Carey et al. (2001) showed that the utilization of both limbs (dominant and non-dominant) was important to soccer performance and that the accuracy rates of dominant and non-dominant limb use were similar. However, in our study with futsal players engaged in elite match play, in which game demands and characteristics are different from soccer (Andrade et al., 2015; Vieira et al., 2016a; Yiannaki et al., 2018), our results were partly compatible with previous laboratory-controlled experiments in which the accuracy rate declined with non-dominant vs. dominant limb use (Barbieri et al., 2008, 2015; Teixeira and Teixeira, 2008; Vieira et al., 2016a).

Although the number of accurate actions from the non-dominant limb was higher than the number of inaccurate actions with the non-dominant limb, players preferred to use the dominant limb to execute those actions that most likely define the game result (i.e., passing and shooting). The correct execution of these actions is apt to be linked to the outcome of the match (Balyan and Vural, 2018), helping to explain why players chose dominant limb performance for important futsal actions. On the other hand, the fact that players had more accurate than inaccurate actions with the non-dominant limb may suggest that encouraging futsal players to use their non-dominant limbs might increase their confidence with this limb and then lead them to use it more frequently without experiencing a non-dominant limb performance decline. Still, player preferences for dominant limb use have merit. Past research has shown, for example, that during the shooting in futsal, there is less performance variability with the dominant (vs. non-dominant) limb (Barbieri et al., 2008), and there is greater cost-effectiveness in dominant movement outputs even though there were no differences found in coordination patterns (Nunome et al., 2006). Thus, futsal players may have a basis for minimal confidence in using the non-dominant limb; using the dominant limb may lead to increased accuracy with ball possession actions.

In fact, it is also important to know the extent of non-dominant skilled limb use during matches and whether player position data might provide insights into dominant and non-dominant limb technical performance and tactical strategies (Lapresa et al., 2013; Gómez et al., 2015b; Sarmento et al., 2016). In contrast to our hypothesis and to findings from previous studies, there were no significant differences in the distance from the goal for accurate vs. inaccurate actions with either the dominant or non-dominant limb. According to Lapresa et al. (2013), ~90% of futsal shots are taken inside the attack zone, suggesting that the location of the court where actions are performed would seem to change its accuracy rate. Also, most of the action sequences that lead to a score start in the defense/midfield zone and end in the attack zone, mainly in the central offensive zone areas (Sarmento et al., 2016), where the percentage of accuracy is higher (Lapresa et al., 2013). In addition, the highest number of effective ball possessions ended in the attack zone in the front of the goal (Gómez et al., 2015b), indicating that the actions performed closest to the goal resulted in a greater accuracy percentage. However, none of these previous studies separated the execution of these actions or ball possessions by limb use (dominant and non-dominant) as did our study. In the context of prior literature, our results suggest that while accuracy of certain technical tasks may vary with whether they occurred in certain court zones, accuracy differences due to distance from the goal are independent of which limb is used. This surprising finding may highlight the unpredictable nature of futsal. Of course, the spatial metric defined here may not have been sufficiently sensitive to detect possible performance asymmetries.

### Right vs. Left-Footed Futsal Players

Our results revealed that right- and left-footed futsal players were not different in the number of ball actions they performed. Also, players with both limb preferences presented higher accuracy rates when using the dominant vs. non-dominant limb. Different from the general population and from soccer athletes in the FIFA world cup 98 (Carey et al., 2001), our cohort of futsal players were ~57% right-footed and ~43% left-footed. A prior study suggested that left-handed athletes may experience an innate neuronal networking advantage relative to right-handed athletes in many sports (Grouios, 2004). Also, Carey et al. (2001) argued *a priori* that relatively “rare” left-footed soccer players may have a strategic advantage in that a defender may be less prepared to duel against left-footed players and a structural advantage in that left-footedness may predispose the player toward a favored use of contralateral visuospatial networks of the right cerebral hemisphere during game-play events. However, in opposition to these theorized performance relationships with upper limb non-dominance (Grouios, 2004), these assumptions may not always hold for lower limb dominance preferences (Carey et al., 2001; Hughes and Wells, 2002). Although additional physiological or anatomic factors may differentiate athletes who prefer to use right and left limbs (Grouios, 2004), they do not seem to meaningfully impact skill-related futsal behavior that was similar in our study for all players, irrespective of their lower limb dominance.

Although our study was conceived using data derived from elite competition, there were important limitations to this study, which need to be considered in further research. First, our data is from 2012 and overall match demands may have changed over time as well as game rules. For example starting from 2020, Reserve Assistant Referee (RAR) is allowed to provide any relevant information regarding the match events to the Referees (FIFA, 2020). We recommend replication studies to update these findings with more recent high-level competitions. Second, we did not consider the total time that the players participated in the games, and this might have influenced our variables of interest. Third, the number of actions performed varied for each limb, possibly affecting our findings. Fourth, the player's lateral preference was not computed considering the gold standard protocol. Fifth, as we have used predominantly knockout stage matches, extrapolations to all stages/competitions should be made with caution. Finally, it is necessary to acknowledge that conclusions regarding the pivots are based on a relatively small sample size. We also recommend that future studies conduct an analysis of additional advanced data that combines technical and spatial metrics (e.g., social network analysis).

Aside from the aforementioned limits, the present study contains some potential implications. For example, an increase in the use of non-dominant limb and its accurate responses could enhance the unpredictability of behavior of the player in-game situations. Thus, training sessions aiming at reducing asymmetries of futsal players would be of overall benefit. Practice constrained to the use of both limbs (in entire court zones) may be also implemented. Although it may represent an impractical analysis to most coaching staff in the real world, recording of the technical actions of the player separately according to the limb used is also advisable based on data presented here (limb-dependent technical performance with ball possession of elite futsal players).

## Conclusion

To conclude it can be stated that when completing technical actions with the ball in futsal, high-level players depended to a greater extent on the use of their dominant lower limb during official matches. This tendency occurred independently of the preferred foot dominance of the players. Excepting a similarity detected between limbs on shooting performance of pivots, players from all positional roles generally showed a higher accuracy rate in receiving, passing, and shooting a ball when using their dominant limb as compared to their non-dominant one during match-play. The use and performance of dominant and non-dominant limbs are independent of the location of the player on the court, and asymmetries in technical tasks with ball possession also exist regardless of the quality of the opponents.

## Data Availability Statement

The raw data supporting the conclusions of this article will be made available by the corresponding author, without undue reservation.

## Ethics Statement

The studies involving human participants were reviewed and approved by UNESP São Paulo State University Júlio de Mesquita Filho Human Research Ethics Committee–Instituto de Biociências, Campus de Rio Claro, under protocol number #4842. Written informed consent for participation was not required for this study in accordance with the national legislation and the institutional requirements.

## Author Contributions

CS, SC, and FB contributed to the conception and design of the study. LV and CS collected and organized the database. CK-F performed the statistical analysis. LV, FB, CK-F, and FS wrote the first draft of the manuscript with critical input regarding intellectual content provided by FC, CS, and SC. All authors contributed to manuscript revision, read, and approved the submitted version.

## Conflict of Interest

The authors declare that the research was conducted in the absence of any commercial or financial relationships that could be construed as a potential conflict of interest.

## Publisher's Note

All claims expressed in this article are solely those of the authors and do not necessarily represent those of their affiliated organizations, or those of the publisher, the editors and the reviewers. Any product that may be evaluated in this article, or claim that may be made by its manufacturer, is not guaranteed or endorsed by the publisher.
