# Supplementary material for: Lateral Preference and Inter-limb Asymmetry in Completing Technical Tasks During Official Professional Futsal Matches: The Role of Playing Position and Opponent Quality
Source: Front Psychol. 2021 Aug 19;12:725097. doi: 10.3389/fpsyg.2021.725097 (PMC8417064; doi:10.3389/fpsyg.2021.725097)
Supplement: Supplementary file 1 [file Table_1.docx]

**Supplementary online Table 1.** Median, interquartile range and extreme values of average Euclidean distance (m) between location where the actions occurred to the goal midpoint using dominant and non-dominant limb.

|  | Dominant Limb |  |  | Non-dominant Limb |  |  | *p-value* |
| --- | --- | --- | --- | --- | --- | --- | --- |
|  | Accurate | Inaccurate |  | Accurate | Inaccurate |  |  |
| Passing (m) | 21.9 (12.1) [12.4 – 24.5] | 23.0 (4.3) [9.9 – 29.9] |  | 21.6 (3.7) [15.1 – 26.3] | 21.6 (9.8) [17.1 – 32.9] |  | 0.83 |
| Shooting (m) | 19.8 (15.3) [5.0 – 41.1] | 20.6 (5.3) [10.3 – 31.6] |  | 20.7 (18.4) [7.2 – 36.4] | 24.6 (13.2) [8.8 – 38.6] |  | 0.83 |
| Ball Reception (m) | 22.0 (2.3) [16.7 – 24.0] | 24.2 (15.8) [12.7 – 36.9] |  | 20.5 (5.7) [16.3 – 26.6] | 17.4 (20.2) [5.6 – 31.9] |  | 0.16 |

*Note:* *p*-value: statistics significance observed through Mann-Whitney’s test. The number of observations were: Dominant with accuracy (Passing: 63, Shooting: 27, Ball Reception: 63); Dominant with inaccuracy (Passing: 49, Shooting: 35, Ball Reception: 24); Non-dominant with accuracy (Passing: 57, Shooting: 19, Ball Reception: 60); Non-dominant with inaccuracy (Passing: 31, Shooting: 19, Ball Reception: 18).
