# Supplementary material for: Lateral Preference and Inter-limb Asymmetry in Completing Technical Tasks During Official Professional Futsal Matches: The Role of Playing Position and Opponent Quality
Source: Front Psychol. 2021 Aug 19;12:725097. doi: 10.3389/fpsyg.2021.725097 (PMC8417064; doi:10.3389/fpsyg.2021.725097)
Supplement: Supplementary file 2 [file Table_2.docx]

|  | Dominant Limb |  |  | Non-dominant Limb |  |
| --- | --- | --- | --- | --- | --- |
|  | Accurate | Inaccurate |  | Accurate | Inaccurate |
| Defenders |  |  |  |  |  |
| Passing | 20.9 (3.2) [10 – 36] | 22.8 (7.6) [13 – 37] |  | 21.0 (4.5) [12 – 29] | 21.0 (7.0) [16 – 34] |
| Shooting | 25.9 (14.5) [5 – 41] | 21.1 (10.3) [8 – 25] |  | 21.1 (10.3) [8 – 25] | 26.9 (16.0) [9 – 39] |
| Ball Reception | 20.3 (4.2) [15 – 26] | 17.9 (15.6) [13 – 35] |  | 22.6 (6.6) [17 – 36] | 21.5 (16.5) [9 – 32] |
| Wingers |  |  |  |  |  |
| Passing | 21.1 (4.1) [12 – 29] | 20.7 (14.4) [7 – 34] |  | 20.9 (5.3) [11 – 27] | 20.6 (12.3) [10 – 39] |
| Shooting | 17.8 (10.1) [7 – 29] | 21.4 (18.7) [9 – 37] |  | 21.4 (18.7) [9 – 37] | 11.8 (23.6) [3 – 35] |
| Ball Reception | 21.8 (3.0) [15 – 25] | 25.6 (11.7) [14 – 37] |  | 21.1 (5.0) [14 – 30] | 21.1 (22.6) [9 – 35] |
| Pivots |  |  |  |  |  |
| Passing | 21.9 (5.3) [18 – 27] | 17.1 (15.3) [9 – 36] |  | 20.7 (5.2) [11 – 33] | 22.7 (17.4) [12 – 34] |
| Shooting | 23.3 (16.2) [15 – 34] | 23.3 (16.2) [15 – 34] |  | 23.3 (16.2) [14 – 33] | 20.0 (21.0) [10 – 30] |
| Ball Reception | 22.0 (2.4) [18 – 26] | 29.0 (6.2) [24 – 32] |  | 21.9 (8.1) [10 – 30] | 32.1 (33.0) [6 – 38] |

**Supplementary online Table 2.** Median, interquartile range and extreme values of average Euclidean distance (m) between location where the actions occurred to the goal midpoint using dominant and non-dominant limb, according to positional role.
