# Supplementary material for: Lateral Preference and Inter-limb Asymmetry in Completing Technical Tasks During Official Professional Futsal Matches: The Role of Playing Position and Opponent Quality
Source: Front Psychol. 2021 Aug 19;12:725097. doi: 10.3389/fpsyg.2021.725097 (PMC8417064; doi:10.3389/fpsyg.2021.725097)
Supplement: Supplementary file 3 [file Table_3.docx]

|  | Dominant Limb |  |  | Non-dominant Limb |  |
| --- | --- | --- | --- | --- | --- |
|  | Accurate | Inaccurate |  | Accurate | Inaccurate |
| Top-ranked |  |  |  |  |  |
| Passing | 21.3 (4.0) [10 – 26] | 21.3 (10.3) [9 – 37] |  | 20.9 (4.9) [11 – 29] | 19.8 (11.4) [10 – 39] |
| Shooting | 21.0 (20.6) [5 – 41] | 20.5 (12.4) [8 – 37] |  | 20.5 (12.4) [8 – 37] | 18.0 (21.9) [3 – 35] |
| Ball Reception | 21.3 (3.1) [15 – 26] | 28.0 (13.4) [14 – 37] |  | 21.1 (6.9) [10 – 29] | 16.5 (25.5) [6 – 38] |
| Middle-ranked |  |  |  |  |  |
| Passing | 21.0 (3.1) [12 – 36] | 20.8 (10.9) [7 – 36] |  | 21.1 (4.8) [12 – 33] | 21.1 (11.3) [12 – 34] |
| Shooting | 17.4 (8.0) [11 – 23] | 22.2 (11.5) [11 – 37] |  | 22.2 (11.5) [11 – 37] | 26.6 (23.0) [9 – 39] |
| Ball Reception | 20.9 (3.1) [15 – 25] | 23.9 (12.5) [13 – 30]† |  | 22.4 (4.5) [16 – 36] | 26.9 (20.6) [9 – 32] |

**Supplementary online Table 3.** Median, interquartile range and extreme values of average Euclidean distance (m) between location where the actions occurred to the goal midpoint using dominant and non-dominant limb, computed according to opponent’s level.

†difference between level of opponent observed through Mann-Whitney’s test (p < 0.05).
